# *Streptococcus agalactiae* Septic Arthritis of the Shoulder and the Sacroiliac Joints: A Case Report

**DOI:** 10.1155/2012/720297

**Published:** 2012-08-13

**Authors:** Yahia Z. Imam, Housam Aldeen Sarakbi, Nagui Abdelwahab, Issa Mattar

**Affiliations:** ^1^General Medicine Division, Department of Medicine, Hamad Medical Corporation, P.O. Box 3050, Doha, Qatar; ^2^Rheumatology Division, Department of Medicine, Hamad Medical Corporation, P.O. Box 3050, Doha, Qatar; ^3^Weill Cornell Medical School, Hamad Medical Corporation, P.O. Box 3050, Doha, Qatar; ^4^Department of Radiology, Faculty of Medicine, Cairo University, Giza, Egypt

## Abstract

Invasive group beta-streptococcal arthritis is being increasingly diagnosed as suggested by recent data. We report a case of a middle-aged lady from Sri Lanka who developed septic arthritis of the right shoulder and the left sacroiliac joint as well as an iliopsoas collection caused by *Streptococcus agalactiae* shortly after labor at Hamad General Hospital in Doha, Qatar. We conclude that *Streptococcus agalactiae* septic arthritis is rare. It can present with invasive disease in adults. It usually targets older females and immuno compromised patients especially those with risk factors for bacteraemia. Therefore a high index of suspicion is needed. Shoulder and sacroiliac joint affection is not uncommon for unknown reasons. Utilizing imaging modalities such as ultrasonography and magnetic resonance imaging is helpful.

## 1. Introduction

Septic arthritis is a rheumatologic emergency requiring prompt diagnosis and treatment. Streptococci are a well-recognized cause of septic arthritis causing up to 20% of all septic arthritides, ranking second to *Staphylococcus aureus* which accounts for 50–60% of cases, while gram-negative rods account for 5–10% [1, 2].

GBS (also known as *Streptococcus agalactiae*) are prominent veterinary pathogens, because they can cause bovine mastitis in dairy cows. The species name “*agalactiae*” meaning “no milk” alludes to this.

Search of the available literature reveals the rare occurrence of Group B streptococci septic arthritis among immuno competent hosts after uncomplicated vaginal delivery.

## 2. Case Report

A previously healthy 45-year-old Sri Lankan housewife, who is gravida 5 and para 5 + 0, was admitted for a normal vaginal delivery at Hamad Medical Corporation. She is known to have *Streptococcus agalactiae* colonization of the vagina as was evident by a high vaginal swab done one month prior to the delivery. The patient developed a persistent right shoulder pain and a low-grade fever 12 hours after delivery of her fifth child via an uneventful noninstrumental vaginal delivery. There was no premature rupture of the membrane, nor were there any laceration in the vagina. She did not receive any peripartum antibiotics. She denied any prior trauma or complaints in the right shoulder or any other joint. She denied having any vaginal discharge. She also had a lower back pain to the extent that she could not walk.

She had an unremarkable past medical and surgical history (no steroid use, autoimmune diseases, diabetes, chemotherapy, or history of HIV infection).

On examination, she was febrile with a temperature of 38.9 Celsius, but otherwise hemodynamically stable. The patient had active arthritis of the right shoulder manifesting with joint swelling, joint tenderness, and marked limitation of movement in all planes.

The patient had also pain over the left sacroiliac joint and a positive Patrick Fabre's test denoting a sacroiliitis.

Blood cultures were drawn and the patient was admitted to the medical ward.

A complete blood count showed elevated white blood cells (WBCs) of 21,000 with neutrophilia, a hemoglobin of 8.9 gm/dL with normochromic normocytic indices, and a normal platelet count. Serum C reactive protein was severely elevated at 314 (normal < 5). Chemistry and liver function test were within normal values.

 Arthrocentesis of the right shoulder attempted blindly on day 1 of the illness produced only 2-3 milliliters of turbid fluid which was only sufficient for a gram staining and culture.

It stained positive for gram-positive cocci in pairs and chains. The patient was started on empirical treatment with ceftriaxone 2 grams intravenously daily.

**Figure 1:**
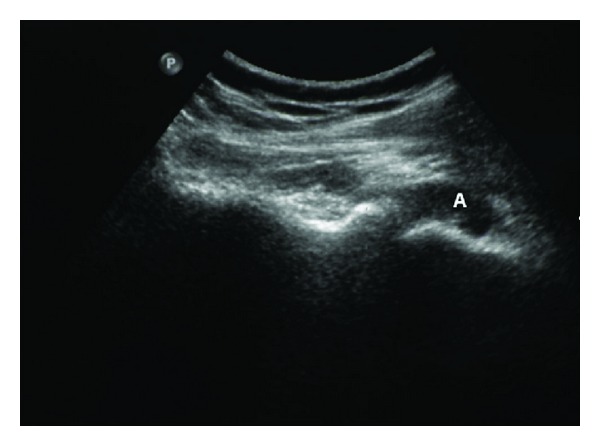
Ultrasound of the right shoulder joint using an axial posterior approach to the glenohumeral articulation showing a markedly distended posterior aspect of joint capsule by fluid (A).

Ultrasonography of the right shoulder on day 2 of the presentation revealed a distended capsule of the shoulder (Figure 1).

Needle aspiration, attempted on day 4 under ultrasonography guidance, came out as a dry tap because of thick pus.

 Culture report from the synovial fluid demonstrated *Streptococcus agalactiae* sensitive to penicillin and ceftriaxone. 2 sets of blood cultures and a high vaginal swap also grew *Streptococcus agalactiae* with similar sensitivity. An echocardiogram was requested and was normal.

The patient was kept on the same antibiotic for ease of administration. She underwent surgical incision and drainage of the right shoulder on day 5. The drainage fluid was turbid but failed to grow any organism. A drain was left in situ, then subsequently removed when no more accumulation was documented via a repeat ultrasound on day 11.

**Figure 2:**
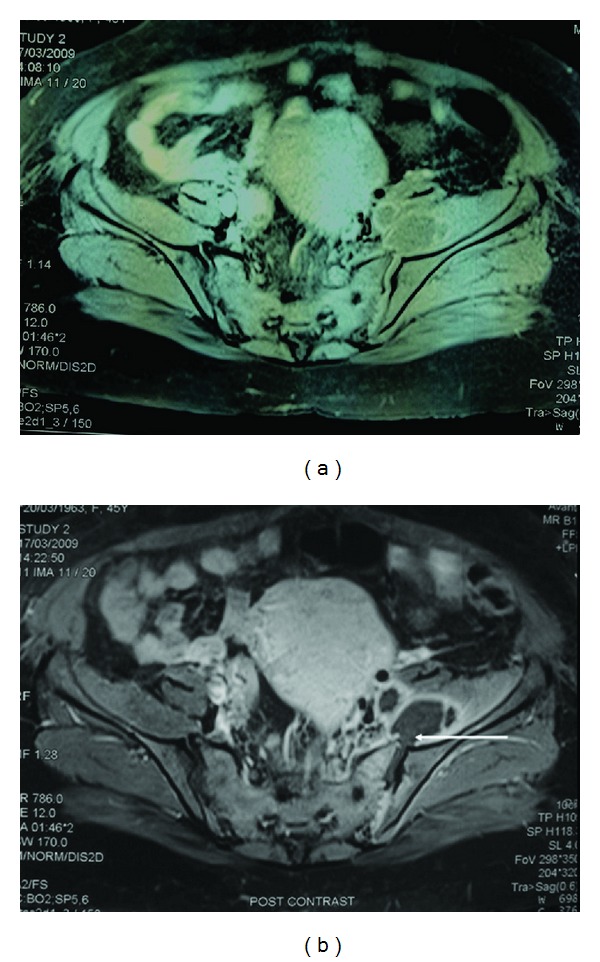
MRI of the pelvis and sacroiliac joints. Coronal T1-weighted (a) as well as (b) fat-saturated (FS) post IV contrast images of the pelvis and sacroiliac joints; showing a 5 cm in diameter multiloculated collection showing dense marginal enhancement deep to left iliopsoas muscle communicating with underlying left sacroiliac joint cavity (see arrow) with evidence of left sacroiliitis in the form of irregular articular surface with subarticular thickening as well as enhancement.

Magnetic resonance imaging (MRI) of the pelvis and the sacroiliac joints (Figure 2) performed on day 10 showed left sacroiliitis with a multiloculated abscess in the anterior superior aspect of the left sacroiliac joint located within the left iliopsoas muscle and extending distally to the level of the left hip joint.

The iliopsoas muscle abscess was drained under radiological guidance on day 12, but the pus also failed to grow the organism; this was attributed to adequate antibiotic coverage.

The patient remained febrile in spite of adequate drainage and 3 weeks of ceftriaxone.

Subsequently the patient was shifted to a once-daily injection of ertapenem. Her fever subsided after 3 days, and she regained some degree of movement in the right shoulder joint.

 She received another 5 weeks of ertapenem and continued to have regular physiotherapy sessions.

**Figure 3:**
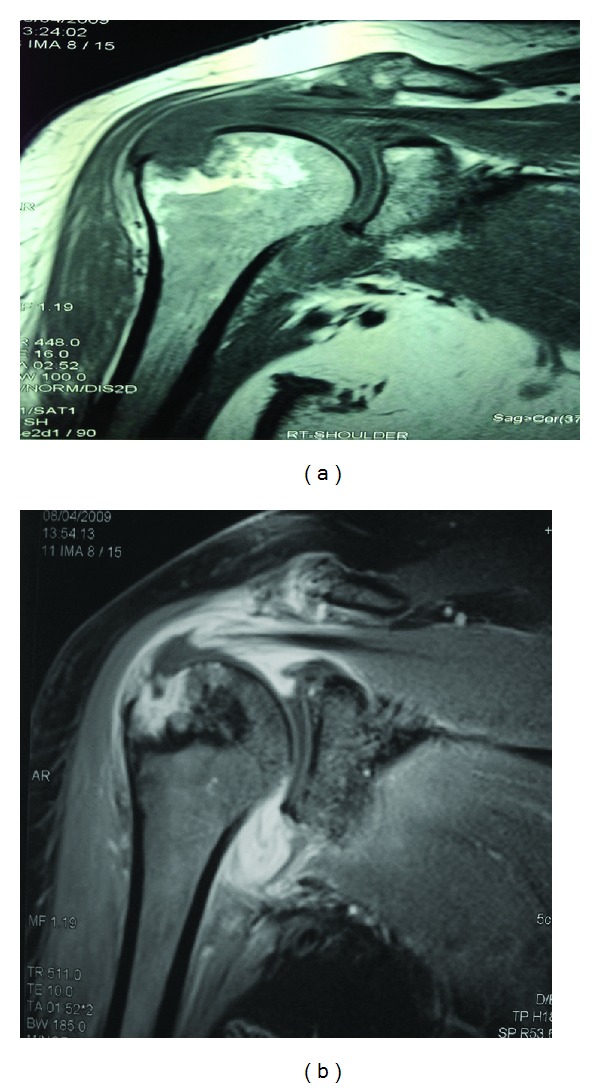
MRI of the right shoulder joint. Coronal T1-weighted (a) and (b) (FS) after IV contrast images of the right shoulder showing dense enhancement of a markedly thickened shoulder joint synovium as well as the subacromial and the subdeltoid bursae with joint effusion is noted. A focal 1 cm area of erosion and underlying trabecular bone enhancement in the greater tuberosity of the humerus and a full thickness tear of supraspinatus tendon with enhancing edges are evident. There is also associated degeneration of the acromioclavicular joint with hypertrophy of its capsule.

After one month of physiotherapy and before the course of antibiotics had finished, there was still limitation of movement of the right shoulder joint raising the possibility of a frozen shoulder, a known complication of septic arthritis; at that time an MRI shoulder showed severe inflammatory process involving the shoulder joint (Figure 3).

The patient had a total course of antibiotic (ceftriaxone + ertapenem) of 8 weeks. She continued with regular physiotherapy for a total of 6 months.

Followup after 8 months reveled pain-free joints with no limitation of movement.

## 3. Discussion

Group B streptococci (GBS) are a common type of the streptococcus bacterium.

Approximately a third of men and women are carriers of GBS in their intestines and a quarter of women carry it in their vaginas. GBS carried in this way can be difficult to detect and does not cause any symptoms [3].

Heavy colonization with this type of bacteria in pregnant women results in adverse outcomes. This includes preterm labor, preterm, and premature rupture of membranes as well as low-birth-weight infants and neonatal disease [4–6].

The current approach to the prevention of Group B streptococcal infection in pregnancy requires intrapartum antimicrobial prophylaxis in term women with microbiological evidence of recent vaginal or rectal Group B streptococcal infection. This is in accordance with the advice from the Center for Disease Control and Prevention (CDC) in 1996 [7].

GBS (also known as *Streptococcus agalactiae*) is now established as a well-known cause of septic arthritis accounting for 5–10% of all septic arthritides [8].

Nolla et al. [9] reported 11 cases over 10 years (1992–2001) where they demonstrated that the organism targeted an older population with those above 60 years of age accounting for 49–91% of cases. They also demonstrated a female preponderance which they attributed to the high rates of vaginal carriage.

Binard et al. [10] reviewed 48 consecutive cases of septic arthritis from May 2000 to May 2004.

Five (10.4%) had arthritis due to Group B streptococci with a mean age of 51.6 ± 18.3 years and a mean hospital stay duration of 13.2 ± 9.23 days.

One of the reported cases was of a 27-year-old lady who developed sacroiliitis postpartum. Specimens from the blood and the vagina showed the same Group B streptococcal strain as in the case of our patient.

Four out of the five cases had either affection of the shoulder, the sacroiliac joints, or both.

In 1984 Small et al. [11] reported 7 cases of septic arthritis caused by *Streptococcus agalactiae*. The shoulder joint was affected in two of them.

A similar case was reported by García et al. [12] where *Streptococcus agalactiae* shoulder arthritis was diagnosed in a 63-year-old female with hepatitis C virus infection.

Similarly Casallo Blanco et al. [13] reported a 47-year-old immunocompetant gentleman with right shoulder arthritis and vertebral osteomyelitis secondary to *Streptococcus agalactiae*.

On the other hand, sacroiliitis was not uncommon as well.

In a review of 13 cases of sacroiliitis caused by *Streptococcus agalactiae* in adults [9, 14–24], there was predominance in the female sex (5 : 1) and the age group from 30 to 40 years. The predisposing factors were in relation to gestation in 4 cases and to cancer of the cervix in another. Other factors implied were a dental handling, a urethral stenosis, and a chronic hepatitis C infection. In 5 cases no factors were identified.

The diagnosis was obtained by means of blood cultures in 12 of the 13 cases and in the remainder by cultivation of the sample obtained by arthrocentesis.

Psoas abscess in a postpartum lady caused by GBS was also reported in the literature [25].

Additionally, this paper emphasizes the contribution of imaging towards the diagnosis. Ultrasonography is a sensitive method for the detection of joint effusions (the hallmark of septic arthritis on ultrasound) in a patient with signs of joint infection before significant lytic lesion in the cartilage or the bone appears as was the case here, It is also helpful for guiding needle aspiration [26].

In the pre-MRI era, imaging of suspected septic arthritis was considered nonspecific. The diagnosis remains a clinical one with the help of arthrocentesis. However, this can be challenging in deep-seated joints such as the shoulder and sacroiliac joints.

MRI can be abnormal as early as 24 hours after the infection, thus becoming a helpful aid to the clinician.

Synovial enhancement, joint effusion (as seen in our patient), and perisynovial edema are typical finding consistent with the clinical diagnosis of a septic joint [27, 28].

## 4. Conclusion

*Streptococcus agalactiae* septic arthritis is a rare complication postpartum. It can present with invasive disease in adults. It usually targets older females and immuno compromised patients especially those with risk factors for bacteraemia.

A high index of suspicion is needed. Shoulder and sacroiliac joint involvement is not uncommon for unknown reasons and should raise the suspicion of invasive GBS disease. Microbiological investigation including synovial fluid and blood cultures is indicated and is often rewarding. Ultrasonography and MRI of the joints are helpful diagnostic tools.
